# New York Odontological Society

**Published:** 1887-06

**Authors:** 


### NEW YORK ODONTOLOGICAL SOCIETY.

REPORTED FOR THE INDEPENDENT PRACTITIONER.

This society convened on the evening of May 10th, at the New York Academy of Medicine.

*Dr. E. A. Bogue*—Cited a case of extensive abrasion of tooth-structure. The patient was present at the meeting, in accordance with a request of Dr. Bogue, and an opportunity was thus afforded for the members to examine the teeth. Gentlemen present were called upon to give some explanation of the case, or to state the cause of such an abraded condition.

*Dr. B. Lord*—Believed it due to severe brushing and the use of coarse, gritty dentifrices. He questioned the patient, who admitted having for a long time used tooth powder containing a large proportion of powdered pumice stone.

*Dr. W. H. Atkinson*—Stated that in regard to the abrasions of these particular teeth he knew nothing, but suspected the tissue at the abraded points to be less dense than in other parts, consequently more readily worn or wasted away. He did not think the brush guilty of causing the mischief. In his judgment, the best method of treating such cases is to cut the structure outside the line of abrasion or soft tissue, shape a good cavity and carefully fill with gold.

*Dr. S. G. Perry*—Said that he had a case similar to the one presented, in which the abrasion was so deep as nearly to expose the pulp of a lower cuspid on the left side of the mouth. As the brush was held in the right hand, and that side was the one most vigorously brushed, he ascribed it to that cause. The patient used "Oriental tooth paste," and Dr. Perry, having observed other cases of abrasion in which this dentifrice had been used, concluded that this might also have had much to do in causing it.

*Dr. W. D. Tennison*—Spoke of a very able and lengthy paper on the subject, read by Prof. Darby, of Philadelphia. He listened very attentively to the essay, hoping to hear something as to the cause of such conditions, but the author himself admitted that he could not explain it. Dr. Tennison could think of no treatment better than that suggested by Dr. Atkinson.

*Dr. E. H. Raymond*—Had a case of extensive abrasion. He carefully prepared cavities and filled with gold, but after a time he observed the same condition reappearing beyond the margin of the filling. He considered it due to disintegration, or to some chemical action which he could not quite comprehend.

*Dr. Bogue*—Remarked that the patient present first consulted him at his office in Paris, stating at the time that he had before been informed that the condition of his teeth was due to acid influences which would ultimately completely encircle his teeth, and destroy them. Dr. Bogue declined to operate for the patient at that time, but the latter recently called at his New York office for further consultation.

*Dr. Perry*—Read a short but very interesting paper, in which he stated that of the many cases in which first permanent molars had been extracted the articulation had been deranged, but he did not consider this little change of so much importance as securing good sound teeth. He thinks that Dr. Davenport (who prepared the paper read at the last meeting) had been so interested in studying articulations that he overlooked the idea of saving by extraction. As regards filing or grinding spaces between teeth, Dr. Perry believes that the practice will condemn itself. He is a firm believer in contour fillings, and would by all means advise the restoration of decayed teeth to their original shape, as far as was practicable. Ten years ago he read a paper on this subject before the New York State Society, at Albany, and after re-reading a few pages of this he declared that the ideas he then entertained on the subject were strongly confirmed by the subsequent ten years of practice. At one period of his professional life he had cut teeth for the purpose of making permanent separations. It was a mistake which he has since greatly regretted. He now works with nature, not against her, and restores teeth to their natural shape whenever this can be accomplished. Where teeth are permanently separated the food will crowd between them, and the gums, which might at first give warning, after a time become callous and allow food to remain and cause decay. If, however, the teeth are knuckled together, each receives support from the others, and thus the food is prevented from becoming forced between them, while there remains space between them near the gum through which fluids might pass and keep them cleansed. He believes "high-water mark" in filling teeth was reached by the late Dr. Varney, and his method has not been surpassed.

The society having previously requested members and friends to bring with them models of mouths from which first permanent molars had been extracted, with a view to compare results, quite a number of casts were presented, some from outside friends, with accompanying letters.

Dr. Taylor, of Hartford, Conn., sent plaster models representing a mouth from which four bicuspids had been removed. They were extracted by advice of the late Dr. J. M. Riggs. The patient had been suffering from severe facial pains, which, from their peculiar character and appearance of the gums, etc., Dr. Riggs diagnosed as exostosis. The teeth were sent with the casts, and their appearance gave evidence of the correctness of the doctor's diagnosis.

Dr. Codman, of Boston, in a letter to the society, states that the first permanent molars should never be extracted until the second molars are fully erupted and the processes are fully formed around them, and until they properly occlude with their fellows, so that as they move forward they will move horizontally and not tip forward.

Dr. L. S. Straw, of Newburgh, sent models of mouths from which the first permanent molars had been removed after the second molars were erupted, showing very fair results.

Dr. Clapp, of Boston, sent casts for inspection ; also a letter of explanation.

Dr. Brackett, of Newport, R. I., sent the model of a mouth from which the first permanent molars had been extracted, showing excellent results.

Dr. Bogue, in commenting on this case, pronounced it a very satisfactory one.

